# Structural and community changes during COVID-19 and their effects on overdose precursors among rural people who use drugs: a mixed-methods analysis

**DOI:** 10.1186/s13722-022-00303-8

**Published:** 2022-04-25

**Authors:** Suzan M. Walters, Rebecca S. Bolinski, Ellen Almirol, Stacy Grundy, Scott Fletcher, John Schneider, Samuel R. Friedman, Lawrence J. Ouellet, Danielle C. Ompad, Wiley Jenkins, Mai T. Pho

**Affiliations:** 1 Department of Epidemiology, New York University School of Global Public Health, New York, NY USA; 2 Center for Drug Use and HIV/HCV Research, New York, NY USA; 3 Department of Sociology, Southern Illinois University, Carbondale, IL USA; 4 Department of Medicine, University of Chicago, Chicago, IL USA; 5 Department of Population Science and Policy, SIU School of Medicine, Springfield, IL USA; 6 The Community Action Place, Inc., Murphysboro, IL USA; 7 Department of Population Health, New York University Grossman School of Medicine, New York, USA; 8 Division of Epidemiology & Biostatistics, School of Public Health, University of Illinois Chicago, Chicago, IL USA

**Keywords:** Overdose, Drug use, COVID-19, Rural, Social determinants of health, Ecosocial theory, Social ecological model

## Abstract

**Background:**

Drug overdose rates in the United States have been steadily increasing, particularly in rural areas. The COVID-19 pandemic and associated mitigation strategies may have increased overdose risk for people who use drugs by impacting social, community, and structural factors.

**Methods:**

The study included a quantitative survey focused on COVID-19 administered to 50 people who use drugs and semi-structured qualitative interviews with 17 people who use drugs, 12 of whom also participated in the quantitative survey. Descriptive statistics were run for the quantitative data. Qualitative coding was line-by-line then grouped thematically. Quantitative and qualitative data were integrated during analysis.

**Results:**

Findings demonstrate how COVID-19 disruptions at the structural and community level affected outcomes related to mental health and drug use at the individual level. Themes that emerged from the qualitative interviews were (1) lack of employment opportunities, (2) food and housing insecurity, (3) community stigma impacting health service use, (4) mental health strains, and (5) drug market disruptions. Structural and community changes increased anxiety, depression, and loneliness on the individual level, as well as changes in drug use patterns, all of which are likely to increase overdose risk.

**Conclusion:**

The COVID-19 pandemic, and mitigation strategies aimed at curbing infection, disrupted communities and lives of people who use drugs. These disruptions altered individual drug use and mental health outcomes, which could increase risk for overdose. We recommend addressing structural and community factors, including developing multi-level interventions, to combat overdose.

*Trial registration* Clinicaltrails.gov: NCT04427202. Registered June 11, 2020: https://clinicaltrials.gov/ct2/show/NCT04427202?term=pho+mai&draw=2&rank=3

**Supplementary Information:**

The online version contains supplementary material available at 10.1186/s13722-022-00303-8.

## Introduction

Overdose rates in the United States have been steadily increasing since 1999, with research identifying four waves of opioid-related overdose deaths. The first wave, from 1999 to 2010, was attributed to prescription opioid pain relievers [1]. The second wave, attributed to heroin, began in 2010 [2]. The third wave, beginning in 2013 was characterized by a sharp increase in overdoses attributed to synthetic opioids, primarily fentanyl and its analogues [3]. Fentanyl, approximately 50 times more potent than heroin [4], greatly increases the risk of fatal overdose for those who consume it, particularly for those unaware that their drug supply contains fentanyl [5]. Fentanyl overdoses can co-occur in persons using stimulants, such as methamphetamine and cocaine, as evidenced by medical examiner and coroner toxicology analyses [6]. This polydrug use represents the fourth wave of the overdose crisis, beginning in 2016, with a steep increase in deaths involving cocaine, methamphetamine, and opioids [7]. The combination of stimulants and opioids can be intentional or unintentional.

Big Events theory suggests that disruptive events like the COVID-19 pandemic can exacerbate rates of drug overdose and infectious disease among drug-using populations due to the disruption of drug markets, behavioral health service delivery, and larger structures such as the economy [8–11]. For example, studies have found that natural disasters such as hurricanes and earthquakes can alter drug markets and increase the likelihood of experiencing withdrawal symptoms [12–14], both of which increase risks for overdose. Indeed, increases in overdoses have occurred during the COVID-19 pandemic [15, 16], particularly in the early months beginning March 2020 and peaking in May 2020, which coincides with nationwide mitigation strategies, particularly stay-at-home orders [17, 18]. Importantly, big events can limit access to harm reduction and healthcare resources that may mitigate overdose risk [12–14]. A recent study of syringe service programs (SSPs) reported substantial closures and service reductions during the COVID-19 pandemic [19]. Other studies have, however, demonstrated ways that some SSPs rapidly pivoted during the pandemic to engage participants in care. Lock boxes and mail-based distributions have been introduced for contactless delivery [20, 21] and, in one case, building enclosures resembling telephone booths were built to facilitate client communication with service providers while maintaining social distancing [22]. Big events that destabilize environments can also harm the psychosocial wellbeing of persons who use drugs and impact their drug use patterns. Since the pandemic, there have been sharp increases in depression and anxiety [23], conditions associated with increased substance use that in turn are likely to increase the risk for overdose [24]. Upticks in drug overdose, anxiety and depression could last long after the pandemic subsides [11, 25, 26].

While COVID-19 and its mitigation efforts likely affect drug use patterns among people who use drugs, those living in rural areas may be disproportionately impacted for at least three reasons. First, many rural areas have higher rates of opioid and methamphetamine use than found in non-rural areas [27]. Second, rural areas often experience resource paucity, poor economic opportunity, decreased availability of drug treatment and harm reduction services, and increased food insecurity and homelessness [28, 29]. Third, people who use drugs in rural areas may experience higher levels of stigma from community members, healthcare providers and law enforcement, which may be exacerbated during COVID-19 and contribute to a greater likelihood of using drugs alone and a reluctance to seek medical care [21, 30–32].

To better elucidate why Big Events have disproportionate impact on communities we draw upon ecosocial theory [33]. A key concept of ecosocial theory is embodiment, which explains how individuals biologically incorporate their social circumstances. For example, exposure to fentanyl can create the biological response of overdose, but the clustering of overdoses in certain communities and regions cannot be explained solely by biology. The disproportionate rates of overdose are better understood through an ecosocial lens, which includes the varying power dynamics embedded in political and economic systems in society that affect both access to resources and exposures to harmful situations. At the same time, health is also shaped by biology, including generational histories such as experiences with discrimination. Lead poisoning offers an example. Lead exposures are disproportionately experienced by lower socio-economic communities and people of color, populations long constrained by multi-level discrimination [34]. Lead exposure experienced during pregnancy can cross the placenta and expose the fetus, creating transgenerational transmission that can further disadvantage these populations [35]. In addition, there is a cumulative interplay between exposure, susceptibility, and resistance.

A final concept in ecosocial theory is how the state creates structures that perpetuate inequities. For example, the criminalization of drug use and the attending stigma create situations that map onto the bodies of people who use drugs and result in poor health outcomes, including overdose [36–38]. The COVID-19 pandemic is a big event that could alter societal conditions, including restricting resources for people who use drugs, disrupting illicit drug markets, and reducing the ability of people who use drugs to act on health supports. These conditions may create further constraints and harsher health outcomes, such as drug overdose.

This study sought to explore how the COVID-19 pandemic affected overdose risk among people who use drugs in rural southern Illinois. We sought to understand how larger forces at the structural and community level influenced individual psychosocial conditions and drug use behaviors, and how people who use drugs embodied these conditions. Guided by socio-ecological [39, 40] and ecosocial theories [35] we define level of influence at the structural, community, and individual levels. Structural level factors are larger societal influences, such as policies, including law enforcement and government programs and regulations. Community level factors refer to “relationships among organizations, institutions, and informal networks within defined boundaries,” [39] and include factors such as quality of care, treatment availability and access, and community norms [41]. Finally, individual factors refer to behaviors, psychosocial factors, and biology [41]. Specifically, we explored how the pandemic altered the physical and social contexts of drug use, the types and methods of drug use [27], and how these factors may influence overdose risk [40, 42]. In other words, we sought to understand overdose as “biological expressions of social relations” [33] which necessitates a multi-level exploration of the social world. Below we discuss the prominent themes that emerged from the data.

## Methods

### Study design

From August 2020–May 2021, we conducted a sub-study embedded in a clinical trial in rural southern Illinois that was part of the multisite Rural Opioid Initiative (ROI) cooperative agreement (see Funding). The first phase of the Illinois site of the ROI focused on understanding rural opioid use and the potential for HIV, HCV and other sexually transmitted infections in nine rural regions of the United States, while the ongoing second phase additionally aims to increase access to harm reduction services including disease screening and overdose prevention.

The sub-study used a mixed-methods concurrent triangulation design consisting of a quantitative survey to gain an overview of COVID-19 experiences and semi-structured qualitative interviews to explore participants’ experiences in more detail with the overall goal of gaining rich knowledge of life experiences and concerns [43–45]. We met monthly to discuss themes emerging from the quantitative and qualitative data so that adjustments to the qualitative interview guide could be made, however, data was not fully integrated until the analysis phase [46]. The survey was comprised of Likert scale questions/statements covering the following domains: (1) access to technology such as computers and the internet, harm reduction supplies, and information, (2) ability to obtain resources, asking questions like, *“I’m confident I have a stable place to stay during this time.”*; (3) drug use associated risk, such as *“The process of getting drugs has been more difficult during this time.”*; (4) local attitudes and responses to COVID-19; (5) health care availability and its relation to drug user stigma; and (6) experiences with law enforcement.

Questions in the qualitative interview guides were open ended, allowing us to garner responses that we may not have been previously hypothesized, and thus allowing participants voices to guide the process [47]. Questions included the following domains: experiences with healthcare providers and SSPs; HIV knowledge and prevention; stigma; fentanyl awareness, knowledge and experiences such as using fentanyl test strips; overdose and naloxone; and general drug-use behaviors. Participants were asked a series of questions relating to overdose. Some examples of these questions are *“Tell me about your experiences with fentanyl?”, “Have* your experiences with fentanyl changed since COVID?”, *“Now I’ll ask about your experience with overdosing, by overdose I mean passed out, turned blue, or stopped breathing from using drugs…Have you ever seen a person overdose?”, “Have you ever overdosed? If yes, can you please tell me what happened?”*, and *“Have you experienced any overdoses (you or someone else) since COVID?”.* Within these domains, we asked about changes following the onset of COVID-19 to further elucidate the quantitative survey by gaining an understanding of *why* and *how* things were happening. For example, we had a COVID-19 specific domain which asked questions such as *“How has your life changed since COVID-19?”, “Do you think people who use drugs are more at risk for COVID-19, please explain why?”* and *“Are you concerned about getting COVID-19? Why or why not?”* Most questions included probes intended to deepen the responses.

### Participants

All sub-study participants (survey and semi-structured qualitative interviews) were drawn from the ongoing Illinois ROI clinical trial that started data collection in 2018. Eligibility requirements for the clinical trial included age of 15 years or older, residence in the 16 southernmost Illinois counties which are largely rural, English-speaking, and self-reported use of opioids for non-medical purposes or injecting any type of drug for non-medical reasons within 30 days prior to the interview. Additional eligibility for the qualitative interview was age 18 years or older and having injected drugs in the last 30 days. Participants in the Illinois ROI clinical trial were recruited via an incentivized respondent-driven sampling approach where participants who completed the study’s survey component were able to refer additional participants [48]. Initial recruits (i.e., seeds) who began the chain referral system were identified two ways. First, we collaborated with a local syringe service program that worked closely with the study staff to identify participants who met the eligibility criteria to begin the RDS cycle. Second, we used a venue-based recruiting method where we would conduct community cookouts in local spaces where we could talk with community members and distribute fliers [49].

Given limitations in recruiting people imposed by the COVID-19 pandemic, participants in this sub-study were selected based on availability and convenience. Using the database of the current clinical trial participants, we contacted and interviewed sub-study participants over the phone. Many of the participants who had previously participated in the clinical trial were unreachable as their phone numbers were no longer in service. Some of these unreachable contacts were facilitated through a collaboration with a local SSP that continued to deliver services through home delivery and a mobile van, and thus maintained contact throughout the pandemic. In a few instances, the SSP dropped off and retrieved phones so that participants could take the survey. In addition, the ongoing clinical trial was recruiting new participants, some of whom were recruited for the sub-study.

Participants for the sub-study qualitative interview most often did so in tandem with the COVID-19 survey. However, five participants who engaged in the qualitative interview did not complete the COVID-19 survey at that time and were later unreachable. Our recruitment sought to optimize diversity in drug of choice, age, gender, and race and ethnicity by prioritizing inviting underrepresented groups to take the survey first. However, data was collected during the first wave of the COVID-19 when we were restricted to interviewing over the phone, therefore, participants had to have a working phone to do the survey and qualitative interview. We achieved diversity in drug of choice, age, gender, and education, however diversity in race and ethnicity was low. This lack of diversity in the sub-study reflects both the larger clinical trial (94% of participants identify as non-Hispanic white) and study area demographics (89% non-Hispanic white) [50].

Participants were compensated for participating in the surveys and qualitative interviews. The clinical trial and COVID-19 survey were approved by the University of Chicago Institutional Review Board (IRB) and the qualitative interview study was approved by the New York University IRB.

### Data analysis

We performed descriptive statistics of the following survey data: individual and structural characteristics, healthcare services, syringe-sharing practices, overdose experiences, and attitudes and perceptions regarding COVID-19. All descriptive findings were conducted using Stata SE software Version 17.0.

Semi-structured qualitative interviews were recorded and immediately transcribed. Each transcribed interview was read for accuracy and assigned to one of two coders for coding [47, 51]. Codes were developed using line-by-line coding and then grouping thematically [47, 52]. The two coders met weekly to discuss codes and other analysis issues. During these meetings emerging themes and any potential coding issues were discussed and resolved. When we reached theoretical saturation, meaning the interviews conducted were producing neither new data, themes or categories in relation to COVID-19, we stopped interviewing [47, 53]. Qualitative interviews ranged in length from forty minutes to 2 h. Qualitative data were processed and analyzed using Dedoose (Version 8.3.17). Pseudonyms are used in presenting the qualitative data.

Once the descriptive statistics were run and the codebook for the qualitative data was solidified, we directly compared results looking at how the quantitative data could provide details to help us understand the qualitative themes and vice versa [46].

## Results

From August 2020-May 2021 50 people who use drugs were administered the COVID-19 survey and 17 semi-structured qualitative interviews. Of the 17 semi-structured qualitative interviews, 12 also participated in the quantitative survey. Table 1 describes the characteristics of the qualitative sample. The average age for the qualitative participants was 40 years old. Nine of the 17 were men and 8 were women. All but one participant identified as non-Hispanic white. Most (n = 16) reported accessing a food pantry or government food supports in the past 6 months and over half (n = 10) reported experiencing homelessness in the last 6 months. Table 2 describes characteristics of those who participated in the COVID-19 survey, which are similar to the qualitative participants as well as the larger clinical trial. Additional file 1: Table S1 provides the COVID-19 survey questions for the qualitative participants who also participated in the survey (n = 12). Additional file 1: Table S2 provides the characteristics and overdose experiences of the qualitative participants who also engaged in the COVID-19 survey (n = 12).

**Table 1:** Semi-structured qualitative interview participant baseline characteristics (n = 17), 2018–2021

	n (%)
Age (mean, SD)	40.4 (SD 8.2)
Age (median, IQR)	39 [33, 45]
Gender	
Male	9 (52.9%)
Female	8 (47.1%)
Race/Ethnicity	
White	16 (94.1%)
Black	1 (5.9%)
Education	
Elementary	3 (17.7%)
Some high school	7 (41.2%)
High school graduate	4 (23.5%)
Some college/technical	2 (11.8%)
Missing	1 (5.9%)
Household income less than $25,000	8 (47.1%)
Homeless (past 6 months)	10 (58.8%)
Accessed food supports (past 6 months)	16 (94.1%)
Healthcare services	
Do you currently see a primary care provider at least once per year	5 (26.3%)
Have you ever had bad experiences with primary care such that you considered not going anymore?	10 (52.6%)
Drug use (past 30 days)	
Inject more than one time in a single sitting, from same solution (past 30-day average amount)	11 (64.7%)
Which of the following places have you injected (past 30 days)	
Your own place	15 (88.2%)
A friend, family member, or acquaintance's place	12 (70.6%)
Dealer's place	10 (58.8%)
Car or other vehicle	10 (58.8%)
On the street	2 (11.8%)
Public or state park	4 (23.5%)
In the woods or other outdoor location	5 (29.4%)
Public restroom or restroom in a store/business	8 (47.1%)
Abandoned building	2 (11.8%)
Barn	1 (5.9%)
Sharing practices (past 30 days)	
Use a syringe/needle that you know used by someone else	4 (23.5%)
Use a cotton/cooker/spoon rinse by somebody else	6 (35.3%)
Let someone else use a cotton/cooker/spoon after used it	4 (23.5%)
Inject drugs that somebody else injected	4 (23.5%)
Any of the above	7 (41.2%)
Overdose risk and prevention	
How many times in the past 30 days have you injected alone? (mean, SD)	19.9 (SD 21.2)
How many times in the past 30 days have you injected alone? [median, IQR]	15 [2, 25]
Do you currently carry naloxone or Narcan with you when you leave the house?	6 (35.3%)
Have you given naloxone or Narcan to anyone you do drugs with?	10 (58.8%)
Have you ever used naloxone or Narcan on someone to reverse an overdose?	8 (47.1%)
Do you currently have naloxone or Narcan with you or at home?	10 (58.8%)
Source of needles/syringes (past 30 days)	
Pharmacy	12 (70.6%)
Syringe or needle exchange program	5 (29.4%)
Overdose experiences	
Ever experienced an overdose	12 (70.6%)
Lifetime number of overdose(s) experienced (mean, SD)	5.6 (SD 8.8)
Lifetime number of overdose(s) experienced [median, IQR]	2 [1, 4]
Witnessed an overdose	15 (88.2%)
Have you ever been trained to recognize and respond to an overdose?	8 (47.1%)
Have you ever called 911 because someone overdosed?	9 (52.9%)
Have you ever gotten an overdose reversal kit or prescription for naloxone or Narcan?	10 (58.8%)
Have you ever used naloxone or Narcan on someone to reverse an overdose?	9 (47.1%)
Do you currently have naloxone or Narcan with you or at home?	10 (58.8%)
How many people you know have died from an overdose in the past 6 months?	11 (64.7%)

**Table 2:** COVID-19 survey participants baseline characteristics (n = 50), 2018–2021

	n (%)
*Demographics*	
Age (mean, SD)	40.4 (SD 10.0)
Gender	
Male	26 (52.0%)
Female	24 (48.0%)
Race/Ethnicity	
White	44 (88.0%)
Black	5 (10.0%)
American Indian/Alaska Native	1 (2.0%)
Latino/Hispanic	0 (0.0%)
Education	
Elementary	7 (14.0)
Some high school	21 (42.0)
High school graduate	16 (32.0)
Some college/technical	5 (10.0)
Missing	1 (2.0)
Homeless (last 6 months)	31 (62.0%)
Accessed Food Supports (last 6 months)	46 (92.0%)
Overdose questions	
Ever experienced an overdose	27 (54.0%)
Lifetime number of overdose(s) experienced (mean, SD)	3.5 (SD 6.1)
Lifetime number of overdose(s) experienced [median, IQR]	2 [1, 3]
Witnessed an overdose	38 (76.0%)
Have you ever been trained to recognize and respond to an overdose?	24 (48.0%)
Have you ever called 911 because someone overdosed?	23 (46.0%)
Have you ever gotten an overdose reversal kit or prescription for naloxone or Narcan?	25 (50.0%)
Have you ever used naloxone or Narcan on someone to reverse an overdose?* (n = 39)	19 (48.7%)
Do you currently have naloxone or Narcan with you or at home?* (n = 39)	26 (66.7%)
How many people you know have died from an overdose in the past 6 months?	2.1 (SD 3.0)

*Not all 50 participants were asked this question given a branching logic for participants enrolled in the UG3 study

In the following section, we report participants’ responses regarding (1) structural-level economic forces such as employment, food insecurity, and housing instability, (2) community-level issues related to stigma that emerged as barriers to healthcare in general, and (3) individual-level mental health challenges and changes in drug use. We present the themes that emerged from the qualitative data with data from the COVID-19 survey to provide a more complete picture of landscape changes due to COVID-19 and how they affected conditions related to overdose as well as overdose experiences. The impact of COVID-19 mitigation strategies in rural settings is addressed throughout.

### Structural economic impact

The rural landscape prior to the pandemic was described in the qualitative interviews as a place of widespread poverty with marginal employment opportunities, made more challenging by lack of transportation. The pandemic laid bare these structural barriers to care and health and overall increased overdose risk for participants. Participants noted increased unemployment, food insecurity, and housing instability due to the pandemic.

#### Employment

Overwhelmingly participants described how loss of employment both before the pandemic and resulting from the pandemic burdened them. Maggie, 53-year-old white woman said:

“There's a lot of layoffs in the mines down here. It's a pretty small town, and like I said it's a poverty-stricken area and the people here, it's been generational for generations, like the third generation now going on, where they've been brought up.”

In the COVID-19 survey (Table 3) less than half (n = 19) of respondents agreed with the statement *“I am confident I can maintain a stable income/stream of money during this time.”* In the qualitative interviews, participants talked about why they were economically insecure. Many participants worked in service industries or in other jobs that required in-person interactions. Closures due to the pandemic made it difficult for participants to find work and thereby created or exacerbated economic strain. For example, when COVID-19 mitigation measures expanded, Eva, a 30-year-old white woman who worked two jobs as a restaurant waitress and fast-food employee, reported that the restaurant closed, and the fast-food restaurant severely reduced her hours. She said,

“I've been through several jobs, just due to the lack of hours. Um, it just wasn't able to sustain my rent and bills with the hours they gave me because they couldn't keep everybody at work. Um, a lot of worry, I guess.”

**Table 3:** COVID-19 survey responses (n = 50)

COVID responses	Agree		Neutral		Disagree		Don’t know	
	n	%			n	%		
Technology Use								
I have had reliable access to cell phone service over the past month	43	86.0	1	2.0	6	12.0	0	0.0
I have had reliable access to the internet over the past month	38	76.0	2	4.0	10	20.0	0	0.0
I have technology that I can use to video chat with others	45	90.0	0	0.0	4	8.0	1	2.0
I can reliably receive mail and deliveries at the place I am living/staying	47	94.0	0	0.0	2	4.0	1	2.0
Accessibility of basic resources								
I'm confident I have a stable place to stay during this time	37	74.0	4	8.0	8	16.0	1	2.0
Most people who use drugs have somewhere they can shelter in place during this time	17	34.0	3	6.0	28	56.0	2	4.0
I'm confident I can maintain a stable income/stream of money during this time	19	38.0	3	6.0	28	56.0	0	0.0
I'm confident I can get enough food for myself during this time	40	80.0	2	4.0	8	16.0	0	
I'm confident I can get enough food for my family or other loved ones who I care for or am responsible for during this time	36	72.0	2	4.0	10	20.0	2	4.0
I'm confident I can get necessities such as: electricity, gas, batteries during this time	37	74.0	3	6.0	9	18.0	1	2.0
Accessibility of health supports								
I'm confident I can access medical care, not including COVID-19 related care, during this time	46	92.0	2	4.0	2	4.0	0	0.0
I'm confident I can still get needed medications (e.g., diabetes; anxiety), not including COVID-19 medications, during this time	35	70.0	3	6.0	10	20.0	2	4.0
I'm confident I can access drug use treatment during this time	38	76.0	4	8.0	7	14.0	1	2.0
I'm confident I can access my local SSP and their services/resources during this time	48	96.0	0	0.0	1	2.0	1	2.0
I'm confident I can obtain naloxone/Narcan during this time	45	90.0	1	2.0	3	6.0	1	2.0
I'm confident I can obtain sterile syringes and injection equipment during this time	48	96.0	0	0.0	1	2.0	1	2.0
Healthcare and stigma								
If someone who uses drugs tests positive for COVID-19, they will be treated unfairly or not given the same amount of attention by medical professionals as other COVID-19 patients	23	46.0	3	6.0	20	40.0	4	8.0
Even if I had COVID-19 symptoms, I would be reluctant to seek healthcare because of previous negative experiences with the medical system	16	32.0	0	0.0	34	68.0	0	0.0
If I end up in the hospital for withdrawal or overdose, I am confident that I will be treated appropriately	22	44.0	7	14.0	19	38.0	2	4.0
Drug use								
The process of getting drugs has been more difficult during this time	33	66.0	2	4.0	14	28.0	1	2.0
I worry I might go into withdrawal in the near future	22	44.0	2	4.0	25	50.0	1	2.0
Most people who use drugs have somewhere they can shelter in place during this time	17	34.0	3	6.0	28	56.0	2	4.0
When I get drugs, I have not been able to follow social distancing recommendations (e.g. staying six feet or more away from others, avoiding crowded places)	24	48.0	3	6.0	22	44.0	1	2.0
The types of drugs I use has changed during this time due to availability	25	50.0	2	4.0	22	44.0	1	2.0
Because of less than normal supply, I feel pressure to share drugs, supplies, and equipment	15	30.0	2	4.0	32	64.0	1	2.0
I am more likely to use drugs alone during this time than I was before	25	50.0	3	6.0	21	42.0	1	2.0
I worry that I will end up with a bad batch of drugs that is dangerous in the near future	28	56.0	10	20.0	11	22.0	1	2.0
Mental health								
I feel more depressed, unmotivated, or defeated during this time than I normally do	27	54.0	2	4.0	21	42.0	0	0.0
I feel lonelier during this time than I normally do	20	40.0	4	8.0	26	52.0	0	0.0
I feel more anxious or on edge during this time than I normally do	38	76.0	2	4.0	10	20.0	0	0.0

When asked what she did to survive she said,

“I had some local organizations help and then, I had some property that was my dad's. I ended up selling it because I had to relocate back down here to [town]…. So I went ahead and sold dad’s property and paid my rent up several months in advance down here.”

There was a gender difference in work where men reported working in construction or other manual jobs that required in-person contact at clients’ homes. People in these occupations explained how work slowed down because *“No one wants you working at their house and stuff”* (Brad, 53-year-old white man). Even though participants wanted work, they also knew that doing so came at a risk, as Justin, a 33-year-old white man said,

“It's a lot harder to find work. The work that is out there, you know, you gotta worry about whether or not you're gonna bring it (SARS CoV-2) home to your family. That's the main thing. That bothers me.”

Participants discussed how economic turmoil and fear of acquiring COVID-19 while working in jobs that required face-to-face interactions caused worry and stress at the individual level, which has been shown to increase drug overdose risk [54, 55].

#### Food insecurity

Of the COVID-19 survey respondents over three-quarters felt confident that during the pandemic they could get enough food for themselves and their families or other loved ones (N = 36). Despite most participants reporting sufficient access to food, our qualitative interviews unearthed the considerable strain in doing so. For example, Lisa a 50-year-old white woman who reported recent homelessness during the pandemic said:

“The food pantries and stuff stopped helping for a while. It was really bad here, people eating out of dumpsters and stuff. But they finally got back into the swing of things. And it's hard to get anywhere here, though, because there's only one place in town actually delivers, or you get food . . .like [on the] third Monday and then the [pantry] that started on a Saturday. But being a small town without transportation. [pause] So they had to find anybody to get you to go, to go anywhere for you because, like, pulling teeth, you have to get the rides, bus if you can… They come twice a day to our town and then, you know, getting back. It's like we leave at 10 in the morning, and we'll have you back in five at night if you're lucky.”

#### Housing

About two-thirds (n = 37) of COVID-19 survey respondents were confident they had a stable place to stay during the pandemic. However, less than half (n = 17) believed that most people who use drugs had somewhere they could shelter in place during the pandemic. Being unstably housed or homeless caused immense stress for participants, especially because rural southern Illinois was experiencing cold winter weather at the time of the COVID-19 shutdowns. Michael, a 30-year-old white man explained,

“It’s not healthy, it’s getting cold outside. I can't stay with these shelters. I'm not a violent person. Let me get that straight. You can look at my record, but because I have possession of a weapon, I can’t stay [in a shelter]. I can't stay at the [shelter name]. This one is full and they like shut it down. They ran out of funds.”

Michael described not being able to secure shelter because of his criminal record but needing help during the first wave of COVID-19. This demonstrates how the criminal justice system further constrains people’s lives. He also told us that even during COVID-19 he cannot stay with his family in the area because “*I'm basically up shit creek without a paddle in my family.”*

### Community: stigma and health service use

Healthcare involvement during the pandemic did not change much for participants, likely because they reported low engagement with healthcare prior to the pandemic. About a quarter of the qualitative participants (n = 5) reported seeing a primary care provider once a year. In addition, over half of qualitative participants (n = 10) reported ever having a bad experience with primary care such that they considered not going anymore. As such, participants tried to avoid healthcare settings and pharmacies because they felt they were treated poorly in the past and they anticipated poor treatment in the future [56].

Many participants discussed experiencing stigma at pharmacies, which served as a barrier to accessing sterile syringes. This is particularly troubling because the majority of the qualitative participants reported accessing syringes from a pharmacy in the last 30 days (n = 12), compared to 29% (n = 5) who accessed syringes from the SSP. Carla told us that purchasing syringes was *“awkward, because whenever you walk up to them you tell them what [you want], they just look at you like, you know, like their eyes is literally burning.”* Later in the interview Carla told us that “*every time”* she purchased syringes she was asked for identification, which is illegal in Illinois [57]. Another participant, Kim, a 46-year-old white woman said *“I always just felt like they were judg[ing]. I've actually been refused syringes at [pharmacy] before.”*

Similar to pharmacies, stigma against drug use was a barrier to other health supports. In some instances, participants did not want to go out into social settings at all. Carla explained:

“You can tell when you're being judged just by the way somebody looks at you by their manner, you know, by their body language, everything. Their body language will tell you everything you need to know about a person, everything and I mean you don't want to be judged for what you do. So therefore, it scares you to go outside because you're scared of being judged. So, you just stay inside.”

Despite the all-encompassing stigma that Carla described, the SSP was a safe place that most participants found welcoming and stigma free [58, 59]. For example, Linda, a 54-year-old white woman who previously purchased syringes at pharmacies said you *don't have to be embarrassed anymore* when describing getting harm reduction supplies at the SSP. The SSP continued providing services throughout the pandemic. Participants were able to access new injecting equipment, naloxone and fentanyl test strips by texting, Facebook messaging, or calling the SSP, or by having a friend do so on their behalf. Once the SSP received a request, the items would be delivered to participants homes or other agreed upon places.

Because the survey required participants to be contactable by phone, it is not surprising that most reported reliable cell phone and internet access over the past month. Access to phones and the internet is not, however, a universally available resource among the larger rural population of people who use drugs [60]. For participants without access to technology during pandemic shutdowns intended to curb the spread of COVID-19, healthcare access likely became even more difficult, as Lisa, a 50-year-old white woman pointed out:

“There's no library open for telehealth visits to happen or public rural area where they have a telephone option”

All of this was exacerbated by the low availability of public transportation in these rural areas where going even a few miles was a barrier for some, as Linda explained:

“A lot of people don't have cars. And I live in [town]. Walmart, like, three miles away from where I live. So, it's kind of hard to get out there.

Similarly, Eva, a 30-year-old white woman, said that going to the doctor was *“just a hassle”* because she did not *“have a driver’s license”* and *“just walk[ed] everywhere.”*

### Individual

Structural and community conditions impacted individuals’ daily lives, including risk factors for overdose. The most salient individual challenges mentioned in the qualitative and survey interviews were increased mental health issues related to anxiety, depression, and loneliness. Participants also discussed adaptations in individual drug use behavior resulting from structural changes caused by the pandemic.

#### Mental health

The changing landscape of the pandemic, coupled with pre-pandemic structural and community issues, had serious individual effects on participants’ mental well-being. Just over three-quarters (n = 38) of the COVID-19 survey respondents felt more anxious or on edge during the pandemic, over half (n = 27) felt more depressed, unmotivated, or defeated, and almost half (n = 20) felt lonelier during the pandemic. Caroline a 33-year-old Black woman who was interviewed in October 2020 explained why:

“It's scary. You know, like just because there's so many people dying, Um, people getting sick and like, I just don't want me and my family or, you know, anybody really, like, it's so sad for all these people just to be dying and people be so careless and not want to wear their mask and get mad at the rules of the, um, you know, all these new rules that we have to follow by and it's just like I just don't understand … it's sad sometimes how the world is, so sad.”

Similarly, Elana a 38-year-old white woman interviewed October 2020 told us,

“I’ll be honest with you, whenever I start to hear COVID talk I walk away, I don’t, I don't know. I just don't want to hear it. I don't like to think about death and disease. And I just said that stuff makes me sad…It brings me down a little bit.”

In addition to increased stress and mental health issues, which have been associated with drug overdose, [24] participants discussed changes in the drug market.

#### Drug use behavioral changes

Participants described how the pandemic changed their everyday drug use behaviors. Often these changes were attributed to larger societal factors that were out of their control, such as changes in where and how they could obtain drugs. For example, over half (n = 33) of COVID-19 survey respondents said the process of getting drugs was more difficult during the pandemic, half (n = 25) agreed that the types of drugs they use has changed due to availability, and over half (n = 28) worried that in the near future they would end up with a bad batch of drugs that would be dangerous.

In addition, half (n = 25) of COVID-19 survey respondents said they were currently more likely to use drugs alone than prior to the pandemic, and nearly half (n = 22) worried they would experience withdrawal in the near future. This is noteworthy given that both using drugs alone and unmanaged withdraw symptoms are associated with an increased risk of overdose [61]. In addition, qualitative interview participants reported injecting in places that might require them to hurry and could be less sanitary, such as in a car, on the street, in public, and in the woods. Importantly, public injection has been associated with greater odds of overdose [62]. Further, about one-third (n = 15) of the COVID-19 survey respondents agreed with the following statement *“Because of less than normal supply, I feel pressure to share drugs, supplies, and equipment”* indicating an increased risk for blood-borne infections such as HCV and HIV.

Joe, a 42-year-old white man expressed being more concerned about overdosing because *“the drugs are stronger.”* Joe, who had personally experienced and witnessed overdoses, recounted an overdose experience that happened during the pandemic. Joe thought he was buying a new formulation of heroin, but the drug he purchased appeared to contain fentanyl. He used the drug in the bathroom, walked outside where his dealer and wife were and collapsed from an overdose. His wife called the police, and emergency medical technicians used naloxone to revive him. Joe said:

“I got done using and I was walking out of the bathroom and next thing I know I was in a bathtub with cops everywhere… I got taken to the hospital and then released a couple hours later.”

Similar to Joe, Michael, a 39-year-old white man recounted an experience with overdose due to fentanyl exposure that happened two years ago. Michael said,

“I thought it was heroin and it was mainly [fentanyl] and it laid me out flat. Killed me. They had to do CPR on me but brought me back.”

Michal said the experience was,

“Horrifying. It was. It was really scary. I actually went to rehab and got clean for a little while, and then I got right back on it…I started doing again. I’m okay with it now. Um, you just gotta be really careful. That stuff will kill you quick and you don’t know what you are getting on the streets either. So, a lot of people around here, a lot of my friends died from it. So it’s scary stuff, and you’re really messing with your life when you play with that stuff. But you just gotta know what you’re doing. Don’t do the whole thing, just do a little bit.”

Michael alludes to the fact that people may not be aware of fentanyl in their drugs, and therefore, they need to use harm reduction techniques such as using small amounts to avoid overdose. However, as Michael states, many people he knows have died, therefore, more tools and techniques to avoid fatal overdose are needed.

Finally, participants noted that obtaining heroin had become more challenging during the pandemic, but that fentanyl was more readily available and cost less. Of note, participants discussed fentanyl *“beans”* or *“buttons”* which were described as *“little capsules”* full of fentanyl. These findings are consistent with another study conducted by our research team, which finds an increase in injecting drugs and fentanyl use during the pandemic. When asked why heroin was hard to find, Michele, a 55-year-old white woman said:

“Because the beans, the fentanyl came around and it lasts longer and it’s cheaper.”

## Discussion

Consistent with Big Events theory, structural and social determinants of health that disadvantage people who use drugs in rural southern Illinois were exacerbated by the pandemic. This outcome is also consistent with ecosocial theory, which posits that larger events and structures drive individual phenomena [63]. Structural conditions were problematic pre-pandemic, as the region was stressed economically and lacked healthcare resources, constraining the ability of people who use drugs to act in their own behalf and affecting their health outcomes. For example, 45.4% of the residents in the study’s region live below 200% of the poverty level and all counties are geographic and/or low-income primary and mental health professional shortage areas [64]. Given that social conditions map onto individual health outcomes, and socioeconomic marginalization is associated with increased drug overdose, more attention should be given to this rural region [65].

Participants discussed lack of employment options broadly and pointed to ways that the pandemic worsened their economic conditions. Along with layoffs for those who had jobs, and a decline in jobs available for the unemployed, the COVID-19 pandemic appeared to exacerbate housing and food insecurity. High rates of food insecurity among rural people who use drugs have been reported elsewhere [66, 67]. A key to ecosocial theory is that health disparities reflect power distributions in society and societal systems, and thus the deficits reported by rural people who use drugs are actually health inequities, since the lack of employment, housing and food options are avoidable [35]. Thus, interventions should target root causes of health inequities associated with overdose, such as providing affordable housing, living-wage jobs, and food supports [68, 69]. Importantly, stable housing that is not contingent on substance use or mental health treatment is effective and does not increase alcohol or drug use [70]. From an economic standpoint, housing-first programs are cost effective [71].

At the community level, stigma was a barrier to health services [31], including accessing new syringes and naloxone at pharmacies [32, 72]. We did not find that the pandemic worsened these conditions since they were so problematic pre-pandemic. For example, many participants did not know that naloxone was available from pharmacies without an individual prescription [73]. Similarly, past research on awareness about pre-exposure prophylaxis for HIV prevention has highlighted how stigmatizing drug use serves as a barrier to obtaining needed prevention information among people who use drugs [74–77]. Stigma may be a barrier to acquiring information about naloxone, which could translate into reduced access at pharmacies. Cost may also be an issue for those who attempt to get it without health insurance [78].

Additionally, transportation challenges in the rural areas, a reflection of socio-economic status and systemic inequities, made accessing services challenging, especially as some services contracted during the pandemic. Participants had noted long bus rides and difficulty obtaining rides to appointments pre-pandemic. Given the lack of transportation, participants often could not leave town to access healthcare and pharmacies where they could be more anonymous and thus more likely to avoid community stigma. Service closures and reduced service hours compounded these problems. Telehealth services hold promise for easing access to medical care, particularly during the pandemic, but for some people who use drugs the closing of public places such as libraries that offered internet connections proved an additional barrier. Access to affordable and accessible transportation and broadband internet are two structural issues that could be altered to improve individual health outcomes. In an exception to decreased availability of healthcare resources during the pandemic, the local SSP—which used a mobile delivery model rather than fixed sites—remained open. Participants could text for services and supplies, and the SSP would leave items at their homes or interact outdoors while wearing masks. Research has pointed to the ways that SSPs can benefit clients’ health [79–81] and can be spaces where social networks develop to support health [76, 77]. This study finds that the SSP was a critical service for people who use drugs and was able to adapt to markedly changed circumstances during a big event.

Participants reported that COVID-19 altered drug markets in ways that contributed to changes in drug use. For example, participants reported that after the pandemic’s onset, some drugs became more difficult to obtain, the likelihood of using drugs alone increased, and their fear of overdose grew due to a large increase in the presence of fentanyl. COVID-19 appeared to shape local drug markets in ways that favored the use of fentanyl even among those who much preferred other drugs. Of particular interest, the qualitative portion of the study unearthed an emerging trend of consuming fentanyl ‘beans’, which during the pandemic became more readily available and cheaper than heroin [82]. In addition, a lack of access to recourses like naloxone reported by study participants increased the risk of fatal overdose.

All these changes impacted participants’ mental health. Considerable anxiety and depression have been found among people who use drugs and attributed to a variety of sources, including the experience of stigma [83, 84] and socio-economic deprivation [85]. Further, increases in mental health conditions since the pandemic have been documented [86–88]. While the COVID-19 pandemic did not appear to increase the stigmatization of study participants from its already high level, the potential for higher levels exists in the disadvantages people who use drugs face in getting vaccinated, affording personal protective equipment, and practicing social distancing. To provide insight into interventions to improve mental health outcomes in light of an infectious disease pandemic, future research should include ecosocial analyses on how social, political, and economic systems impact substance use and mental health under “big event” conditions [89].

This study had several limitations. First, the study took place in an overwhelmingly white area, which is reflected in our sample. The COVID-19 pandemic has disproportionately burdened communities of color, particularly Black and Hispanic communities [90, 91] and many rural communities have substantial racial and ethnic minority populations [92, 93]. The possible interactions between the factors described here with racial and ethnic minority status in rural areas needs specific exploration. Research has also found disparities in psychosocial stress by race and ethnicity, with Hispanic communities experiencing greater levels [94, 95]. Future research is needed that includes larger proportions of Black and Hispanic people who use drugs so that their experiences can be assessed. Second, surveys and interviews were done over the phone, which eliminated the ability to talk with people who lacked access to a phone.

## Conclusion

In conclusion, this study found that the COVID-19 pandemic destabilized societal factors at the structural and community levels, which mapped onto bodies at the individual-level, increasing risk of overdose. To reduce overdose risk at the individual level, larger systemic issues need to be addressed, including greater access to economic opportunities that include a living wage, housing, food, transportation, and communication technologies. In addition, community stigma related to drug use should be targeted. Finally, creating multi-level interventions that target all societal levels may be most effective.

## Supplementary Information

**Additional file 1.**Table S1. Participants COVID-19 survey responses (n = 12) of those who completed a qualitative interview. **Table S2.** Participants who completed the COVID-19 survey and qualitative interview (n = 12).

## Data Availability

Portions of the data may be made available upon reasonable request.

## Abbreviation

SSP: Syringe service program
