# Supplementary material for: Structural and community changes during COVID-19 and their effects on overdose precursors among rural people who use drugs: a mixed-methods analysis
Source: Addict Sci Clin Pract. 2022 Apr 25;17:24. doi: 10.1186/s13722-022-00303-8 (PMC9037978; doi:10.1186/s13722-022-00303-8)
Supplement: Supplementary file 1 — Additional file 1.Table S1. Participants COVID-19 survey responses (n = 12) of those who completed a qualitative interview. Table S2. Participants who completed the COVID-19 survey and qualitative interview (n = 12). [file 13722_2022_303_MOESM1_ESM.docx]

| **Table S1. Participants COVID-19 survey responses (n=12) of those who completed a qualitative interview** | | | | | | | | |
| --- | --- | --- | --- | --- | --- | --- | --- | --- |
|  | **Agree** | | **Neutral** | | **Disagree** | | **Don’t Know** | |
| **COVID Responses** | n | % | n | % | n | % | n | % |
| **Technology Use** |  |  |  |  |  |  |  |  |
| I have had reliable access to cell phone service over the past month. | 10 | 83.3 | 1 | 8.3 | 1 | 8.3 | 0 | 0.0 |
| I have had reliable access to the internet over the past month. | 8 | 66.7 | 0 | 0.0 | 4 | 33.3 | 0 | 0.0 |
| I have technology that I can use to video chat with others. | 11 | 91.7 | 0 | 0.0 | 1 | 8.3 | 0 | 0.0 |
| I can reliably receive mail and deliveries at the place I am living/staying. | 12 | 100.0 |  |  |  |  |  |  |
| **Accessibility of Basic Resources** |  |  |  |  |  |  |  |  |
| I'm confident I have a stable place to stay during this time. | 9 | 75.0 | 2 | 16.7 | 1 | 8.3 | 0 | 0.0 |
| Most people who use drugs have somewhere they can shelter in place during this time. | 2 | 16.7 | 1 | 8.3 | 9 | 75.0 | 0 | 0.0 |
| I'm confident I can maintain a stable income/stream of money during this time. | 2 | 16.7 | 2 | 16.7 | 8 | 66.7 | 0 | 0.0 |
| I'm confident I can get enough food for myself during this time. | 9 | 75.0 | 1 | 8.3 | 2 | 16.7 | 0 | 0.0 |
| I'm confident I can get enough food for my family or other loved ones who I care for or am responsible for during this time. | 8 | 66.7 | 1 | 8.3 | 3 | 25.0 | 0 | 0.0 |
| I'm confident I can get necessities such as: electricity, gas, batteries during this time. | 7 | 58.3 | 2 | 16.7 | 3 | 25.0 | 0 | 0.0 |
| **Accessibility of Health Supports** |  |  |  |  |  |  |  |  |
| I'm confident I can access medical care, not including COVID-19 related care, during this time. | 10 | 83.3 | 1 | 8.3 | 1 | 8.3 | 0 | 0.0 |
| I'm confident I can still get needed medications (e.g., diabetes; anxiety), not including COVID-19 medications, during this time. | 6 | 50.0 | 2 | 16.7 | 4 | 33.3 | 0 | 0.0 |
| I'm confident I can access drug use treatment during this time. | 8 | 66.7 | 1 | 8.3 | 3 | 25.0 | 0 | 0.0 |
| I'm confident I can access my local SSP and their services/resources during this time. | 11 | 91.7 | 0 | 0.0 | 1 | 8.3 | 0 | 0.0 |
| I'm confident I can obtain naloxone/Narcan during this time. | 11 | 91.7 | 0 | 0.0 | 1 | 8.3 | 0 | 0.0 |
| I'm confident I can obtain sterile syringes and injection equipment during this time. | 11 | 91.7 | 0 | 0.0 | 1 | 8.3 | 0 | 0.0 |
| **Healthcare and stigma** |  |  |  |  |  |  |  |  |
| If someone who uses drugs tests positive for COVID-19, they will be treated unfairly or not given the same amount of attention by medical professionals as other COVID-19 patients | 6 | 50.0 | 2 | 16.7 | 4 | 33.3 | 0 | 0.0 |
| Even if I had COVID-19 symptoms, I would be reluctant to seek healthcare because of previous negative experiences with the medical system | 4 | 33.3 | 0 | 0.0 | 8 | 66.7 | 0 | 0.0 |
| If I end up in the hospital for withdrawal or overdose, I am confident that I will be treated appropriately | 4 | 33.3 | 3 | 25.0 | 5 | 61.7 | 0 | 0.0 |
| **Drug Use** |  |  |  |  |  |  |  |  |
| The process of getting drugs has been more difficult during this time. | 9 | 75.0 | 0 | 0.0 | 3 | 25.0 | 0 | 0.0 |
| I worry I might go into withdrawal in the near future. | 9 | 75.0 | 0 | 0.0 | 3 | 25.0 | 0 | 0.0 |
| Most people who use drugs have somewhere they can shelter in place during this time. | 2 | 16.7 | 1 | 8.3 | 9 | 75.0 | 0 | 0.0 |
| When I get drugs, I have not been able to follow social distancing recommendations (e.g. staying six feet or more away from others, avoiding crowded places). | 9 | 75.0 | 0 | 0.0 | 3 | 25.0 | 0 | 0.0 |
| The types of drugs I use has changed during this time due to availability. | 7 | 58.3 | 0 | 0.0 | 5 | 41.7 | 0 | 0.0 |
| Because of less than normal supply, I feel pressure to share drugs, supplies, and equipment. | 6 | 50.0 | 0 | 0.0 | 6 | 50.0 | 0 | 0.0 |
| I am more likely to use drugs alone during this time than I was before. | 8 | 66.7 | 0 | 0.0 | 4 | 33.3 | 0 | 0.0 |
| I worry that I will end up with a bad batch of drugs that is dangerous in the near future. | 9 | 75.0 | 0 | 0.0 | 3 | 25.0 | 0 | 0.0 |
| **Mental Health** |  |  |  |  |  |  |  |  |
| I feel more depressed, unmotivated, or defeated during this time than I normally do. | 9 | 75.0 | 1 | 8.3 | 2 | 16.7 | 0 | 0.0 |
| I feel lonelier during this time than I normally do. | 7 | 58.3 | 1 | 8.3 | 4 | 33.3 | 0 | 0.0 |
| I feel more anxious or on edge during this time than I normally do. | 11 | 91.7 | 1 | 8.3 | 0 | 0.0 | 0 | 0.0 |

| **Table S2. Participants who completed the COVID-19 survey and qualitative interview (n=12)** | | |  |
| --- | --- | --- | --- |
| **Demographics** | N (%) | | |
| **Age** (mean, SD) | 42.8 (SD 8.4) | | |
| **Gender** |  |  | |
| Male | 5 (51.7%) | | |
| Female | 7 (58.3%) | | |
| **Race/Ethnicity** |  |  | |
| White | 11 (91.7%) | | |
| Black | 1 (8.3%) | | |
| **Education** |  |  | |
| High school diploma or GED | 1 (8.3%) | | |
| Some college | 1 (8.3%) | | |
| Associate degree, trade, or technical school | 4 (33.3%) | | |
| Completed degree | 1 (8.3%) | | |
| Declined/Missing | 5 (41.7%) | | |
| **Homeless (last 6 months)** | 7 (58.3%) | | |
| **Income (less than $25k)** | 8 (66.7%) | | |
| **Overdose Questions** |  | | |
| Ever experienced an overdose |  | | |
| Lifetime number of overdose(s) experienced (mean, SD) | 6.2, SD 10.3 | | |
| Lifetime number of overdose(s) experienced [median, IQR] | 1.5 [1, 4] | | |
| Witnessed an overdose | 12 (100.0%) | | |
| Have you ever been trained to recognize and respond to an overdose? | 8 (67.7%) | | |
| Have you ever called 911 because someone overdosed? | 7 (58.3%) | | |
| Have you ever gotten an overdose reversal kit or prescription for naloxone or Narcan? | 8 (66.7%) | | |
| Have you ever used naloxone or Narcan on someone to reverse an overdose? | 6 (50.0%) | | |
| Do you currently have naloxone or Narcan with you or at home? | 8 (66.7%) | | |
| How many people you know have died from an overdose in the past 6 months? (mean, SD) | 3.7, SD 5.3 | | |
